# Comparison of OvaCyte™ Speciation and PNA staining for the detection of *Haemonchus contortus* in ovine faecal samples

**DOI:** 10.3389/fvets.2025.1688644

**Published:** 2025-12-10

**Authors:** Nagwa Elghryani, Geetika Lahan, Jayanta Bor Gohain, Trish McOwan, Theo de Waal

**Affiliations:** 1 School of Veterinary Medicine, University College Dublin, Dublin, Ireland; 2 Telenostic Ltd., Kilkenny, Ireland; 3 Department of Biology, Faculty of Arts and Sciences-Gamines, University of Benghazi, Benghazi, Libya

**Keywords:** ovine, parasitic diagnostics, faecal egg count, OvaCyteTM Speciation, *Haemonchus contortus*

## Abstract

**Background:**

*Haemonchus contortus* is one of the most pathogenic and economically important species of gastrointestinal nematodes (GINs) affecting livestock. Recent advancements have led to the development of an artificial intelligence (AI)-powered OvaCyte™ Speciation method, which can quantify the proportion (%) of *Haemonchus contortus* eggs among strongyle eggs in a faecal sample.

**Methods:**

This study evaluated the diagnostic performance of the OvaCyte™ Speciation method compared to the conventional peanut agglutinin (PNA) fluorescence staining method as a true reference across 110 fresh ovine faecal samples.

**Results:**

Of the total samples, 92 (82.9%) tested positive for *Haemonchus contortus.* Based on the OvaCyte™ Speciation egg counts, the results showed a marked increase in the mean *Haemonchus contortus* egg count that corresponded with rising mean strongyle egg counts. A very strong correlation was observed between OvaCyte™ Speciation and peanut agglutinin staining (*rₛ* = 0.90, *p* < 0.05). OvaCyte™ Speciation demonstrated a high sensitivity of 100% and a specificity of 89% for the detection of *Haemonchus contortus*.

**Conclusion:**

These findings validate OvaCyte™ Speciation as a reliable alternative to the peanut agglutinin staining method, particularly in field and clinical settings where rapid turnaround and ease of use are critical. Its application could enhance routine herd surveillance and enable evidence-based parasite control in resource-limited or on-farm environments.

## 1 Introduction

*Haemonchus contortus* is one of the most important gastrointestinal nematodes (GINs) that causes severe losses in small ruminants, particularly in sheep and goats (1, 2). Infections can lead to considerable financial losses due to reduced productivity and treatment costs, with annual economic burdens reported in millions of euros across different regions (3).

*Haemonchus contortus* feeds on blood in the abomasum. Clinical haemonchosis results primarily from this blood loss, manifesting in three forms: hyperacute, acute and chronic (4). These forms can range from sudden death due to haemorrhage to prolonged anaemia, lethargy, bottle jaw and weight loss (4). Within the broad diagnostic landscape of parasitology for *H. contortus* identification, each diagnostic approach occupies a distinct niche. Traditional microscopic methods include manual microscopy, in which *H. contortus* eggs are manually counted by an expert parasitologist, and coproculture, where the infective L3-stage larvae of *H. contortus* are identified (5). Another diagnostic procedure involves fluorescent lectin staining using peanut agglutinin (PNA). PNA binds specifically to carbohydrate residues on the eggshells and larvae of *H. contortus*, enabling its distinction from other *trichostrongylid* species under a fluorescent microscope (6). In addition, molecular techniques such as quantitative polymerase chain reaction (qPCR) and droplet digital polymerase chain reaction (ddPCR) have been used on faecal samples to detect the presence of *H. contortus* and other strongyle species (7–9). Table 1 provides an overview of diagnostic methods for GIN detection, summarising their key features, performance, and practical applications.

**Table 1:** Comparison of diagnostic methods for detecting gastrointestinal nematodes in small ruminants, including their principles, quantitative capability, processing time and field suitability.

Method	Principle	Quantitative capability	Time	Field use	Equipment requirement	Training requirement
Manual Microscopy (5, 32)	FEC-Morphological ID of eggs	Yes	1 h at the most	Low	Microscope with high-quality optics, glass slide/McMaster egg counting chamber/ Mini-FLOTAC disc	High: Level training required
Larval Culture and Morph ID (5, 28)	Culture to L3, morphological ID	No	7–10 days	Very low	Incubator, microscope, slide, coverslips, iodine stain, beaker, Baermann test equipment	High: Full morphology training and months of experience required
Fluorescent staining with PNA (6, 28)	Species-specific detection of *H. contortus*	Semi	2–3 h	Low	Fluorescence microscope, PNA stain, incubator, tubes, vortex, centrifuge	High: Complex training required
Quantitative PCR (28)	Species-specific DNA detection	No	2–4 h	Very low	Real-time PCR machine, micropipettes, PCR tubes/plates, centrifuge, vortex, cold storage	High: Molecular lab experience required
ddPCR (8)	Direct droplet, species-specific DNA detection	Yes	2–4 h	Very low	Droplet digital PCR system, droplet generator, thermal cycler, pipettes, consumables	High: advanced molecular biology training required
AI-based Image Analysis-OvaCyte™ (13)	FEC-Deep learning on egg morphology	Yes	< 20 min	High	OvaCyte™ analyser, single-use OvaCyte™ Plus test kit (cassette and flotation solution)	Customer-friendly

In parasitology, faecal egg counts (FECs) are particularly valuable for diagnosing *H. contortus* infections due to high fecundity of the female worm (5,000–12,000 eggs/day) (5). This contrasts with other strongyles, such as *Teladorsagia circumcincta*, which produces ~400 eggs/day. Consequently, high FECs without diarrhoea are strong indicators of *H. contortus* infection (6). However, in cases of mixed infections, which are commonly observed, total strongyle egg counts lack specificity, necessitating methods that allow for species-level identification.

In addition to parasitology, there are several other tools and diagnostic methods used to diagnose haemonchosis, particularly in field or clinical settings (10). These methods often rely on the physiological effects of the parasite, particularly anaemia—for example, FAMACHA© system—or molecular/serological markers such as PCR and enzyme-linked immunosorbent assay (ELISA) (10, 11). However, the majority of these methods are not specific to *H. contortus*, as other factors causing anaemia may confound the results.

The diagnostic methods described above for the detection of *H. contortus* infection remain constrained by the need for laboratory infrastructure, skilled technicians, and lengthy processing times, limiting their suitability for on-farm decision-making. The OvaCyte™ Speciation system addresses these gaps by providing a rapid, automated, artificial intelligence (AI)-powered platform capable of accurate species-level identification and quantification of clinically significant helminth and coccidia eggs/oocysts, with applications in laboratory, veterinary and point-of-care settings. Currently, the system can detect nematodes, cestodes, trematodes, and coccidia in faecal samples from multiple host species, including equine, bovine, ovine, canine, and feline. A 2020 study by Elghryani et al. (12) validated OvaCyte™ for use in bovine faecal samples, demonstrating consistent egg counts when compared to the McMaster and Mini-FLOTAC techniques. Building on this, a 2023 study (13) evaluated the performance of OvaCyte™ in equines by comparing it with the McMaster and Mini-FLOTAC methods. This study emphasised the detection of strongyle-type eggs, which were the most frequently observed, and found a strong correlation and agreement between the OvaCyte™ analyser and the traditional techniques. Using Bayesian latent class analysis, OvaCyte™ exhibited the highest sensitivity (Se) for detecting strongyles, *Parascaris* spp., and *Anoplocephala* spp., reinforcing its utility as a reliable diagnostic tool in equine parasitology. Recently, the OvaCyte™ Pet Analyser was further assessed in a recognised study that assessed its diagnostic performance in companion animals. The study aimed to estimate its effectiveness by comparing results with established benchmarks used in reference laboratories. A total of 141 canine faecal samples, each containing at least one parasite species identified via double centrifugation, were tested using four index tests: centrifugal flotation (with 1 g and 2 g faecal samples), passive flotation, and the OvaCyte™ technique. The OvaCyte™ Pet Analyser demonstrated high Se (90 to 100%) across various parasite species. Its performance for detecting roundworms and hookworms was significantly higher compared to both the 1 g centrifugal and passive flotation methods (*p* < 0.05). In addition, it achieved 100% Se and 89% specificity (Sp) in detecting *Capillaria* spp., significantly outperforming all other flotation techniques (*p* < 0.001).

The advancement of the OvaCyte platform to include OvaCyte™ Speciation for distinguishing *H. contortus* from other nematode species offers the potential to integrate AI-based tools, providing an affordable and scalable solution for ongoing surveillance and clinical diagnostics (14, 15). These results underscore the variability in diagnostic accuracy across methods and highlight OvaCyte™ Speciation’s potential as a more sensitive and specific tool for parasite detection in veterinary practice. This study aimed to assess the correlation between a laboratory-based PNA fluorescence method and the OvaCyte™ Speciation point-of-care system in estimating the proportion of *H. contortus* eggs among strongyle-type eggs in ovine faecal samples.

## 2 Methods

### 2.1 Introduction to the methods

OvaCyte™ Speciation (Ovine) is an AI-driven system designed for rapid morphometrical analysis of GIN eggs (16). This includes analytical features such as symmetry, major and minor axes, and aspect ratio. All required equipment is supplied with the system, including the OvaCyte™ analyser and the single-use OvaCyte™ Plus test kit (cassette and flotation solution). Sample preparation takes less than 2 min, and the automated analysis is completed in <20 min.

Fluorescent microscopy involves staining *H. contortus* eggs with PNA, a fluorescent lectin stain that selectively binds to egg surface glycoproteins. Required equipment includes a fluorescence microscope, PNA stain, an incubator, a vortex and a centrifuge. The procedure is semi-quantitative and involves manual counting of fluorescently labelled *H. contortus* eggs within the total strongyle egg population. Each sample requires approximately 90 min of technician time.

### 2.2 Sample collection and management

A total of 110 fresh ovine faecal samples were collected from mixed flocks across various farms and marts in Kilkenny, Ireland, in May and June 2025. Faecal samples were selected randomly from freshly deposited faeces within each flock, gathered from the ground, aliquoted into labelled pots weighing approximately 30 g and sent directly to University College Dublin (UCD), where diagnostic tests were carried out for the detection of *H. contortus* eggs. The two diagnostic methods mentioned above were performed.

### 2.3 Sample preparation

Initially, each sample was thoroughly homogenised with a spatula for the uniform distribution of parasite eggs. As described by Elghryani et al. (13), 3 g of faeces was mixed with 42 mL of saturated sodium chloride solution (specific gravity 1.2) and sieved through a 212 μm wire mesh filter.

#### 2.3.1 OvaCyte™ Speciation method

The prepared filtrate from each sample described above was injected into the OvaCyte™ Plus cassette, following the standard operating procedure (13). The cassette was scanned on the OvaCyte™ analyser, which involved an active flotation process (shaking) and subsequent image capture. After the images were uploaded and analysed using the custom OvaCyte™ Speciation models, the proportion (%) of *H. contortus* eggs among the strongyle eggs for each sample was recorded. *H. contortus* is represented as an “estimation” of the percentage of the total strongyle eggs (where the full egg is visible in the frame). The cut-off detection limit is approximately 3 epg for strongyles. However, a higher cut-off of 50 epg was applied for the detection of *H. contortus*. These cut-off values were designed to optimise the sensitivity of the overall test for the detection of strongyles at very low levels while ensuring high specificity in the estimation of *H. contortus.* The applied 50 epg threshold reduces the likelihood of false-positive classification at low egg counts, where morphological overlap between species can compromise prediction accuracy. For example, at 50 epg, approximately 15 eggs would need to be misclassified as *H. contortus* to affect the result, compared to only one egg at a 3 epg threshold. Consequently, this higher cutoff improves the robustness of species-level estimation without materially affecting clinical interpretation.

### 2.3.2 PNA method

#### 2.3.2.1 Egg isolation

Egg isolation was performed using the filtrate in the OvaCyte™ Plus cassettes. After the completion of the OvaCyte™ Speciation analysis for each sample, the filtrate was washed from the cassette and transferred into a conical flask. To ensure maximum egg recovery, the channel of the cassette was subsequently backflushed with distilled water into the same flask. The volume in the flask was then brought up to 250 mL with distilled water and allowed to sediment undisturbed for 15 min (17). The supernatant was carefully decanted until the volume was reduced to 150 mL. The flask was refilled to the original volume with distilled water and left to sediment. After 15 min, the supernatant was discarded and the sediment containing the eggs was collected in a 10 mL plastic conical tube (Sarstedt, Australia) using a plastic pipette (Wheaton, China). The tube was then filled with 3 mL of phosphate-buffered saline (PBS), pH 7.4 (Sigma, P5493-1 L) and centrifuged at 180 x g for 5 min. The supernatant was removed, and the sediment was resuspended in 1.5 mL of PBS.

#### 2.3.2.2 Lectin staining process

The egg suspension was stained by adding 10 μL of PNA lectin (Sigma, Cat. No. L-7381; derived from *Arachis hypogaea,* diluted in deionised water at 2 mg/2 mL). As described (12), the mixture was vortexed briefly and then incubated for 1 h at 20 °C ± 2 °C, with an additional vortex after 30 min. After incubation, 3 mL of PBS was added to the mixture, followed by centrifugation at 180 x g for 5 min. The supernatant was removed, and the pellet was washed again with PBS in the same manner. The fluid was removed until approximately 0.5 mL of the final egg sediment remained. *H. contortus* eggs were counted by first thoroughly mixing the egg sediment, then placing 10 μL onto a glass slide, covering it with a coverslip and examining it under a fluorescence microscope (Nikon CoolLED pE-300) Controller with FITC filter (Excitation: 465–495 nm, Dichroic Mirror: 505 nm, Bandpass: 515–555 nm, United Kingdom). To count strongyle eggs, the same slide was observed under an Optika microscope (model B-800BF, Ponteranica, Italy). This process was repeated at least three times or more, using fresh aliquots each time, until the total strongyle egg count exceeded 50. For the samples that tested negative for *H. contortus* or had fewer than 50 strongyle eggs, the entire aliquot was examined, and the total counts were recorded. All samples were examined by an expert parasitologist at magnifications of 100x and 200x. To determine the percentage of *H. contortus* eggs, the number of *H. contortus* eggs was divided by the total number of strongyle eggs detected.

### 2.4 Statistical analysis

Statistical analysis was performed using R (version 4.4.2) (18) to analyse the correlation between the OvaCyte™ Speciation and PNA methods. The normality of both datasets was assessed using the Shapiro–Wilk test. As the PNA data did not follow a normal distribution, a non-parametric Spearman’s rank correlation (*r_s_*) was used to evaluate the association between the two methods.

Diagnostic Se and Sp were calculated using the PNA method as the reference (gold standard). The 95% confidence interval (CI) was calculated in Microsoft Excel (Version 2,502) using the Clopper-Pearson method for binomial proportions. Samples with *H. contortus* counts below 50 epg on OvaCyte™ Speciation were classified as either positive or negative based on their corresponding classification in the PNA reference method. As a detection limit cannot be determined with the PNA method for the purpose of comparison (it does not provide a reliable epg), this approach was adopted. The classification rule is clarified in Table 2.

**Table 2:** Binary classification of OvaCyte™ Speciation under three categories [positive >50 egg per gram (epg), positive <50 egg per gram (epg) and negative] against the peanut agglutinin (PNA) method for the detection of *H. contortus.*

Methods		OvaCyte™ Speciation		
		Positive >50 epg	Positive <50 epg	Negative
PNA	Positive	TP	TP	FN
	Negative	FP	TN	TN

TP is true positive, TN is true negative, FN in false negative and FP is false positive, based on the PNA reference method.

## 3 Results

**Figure 1:**
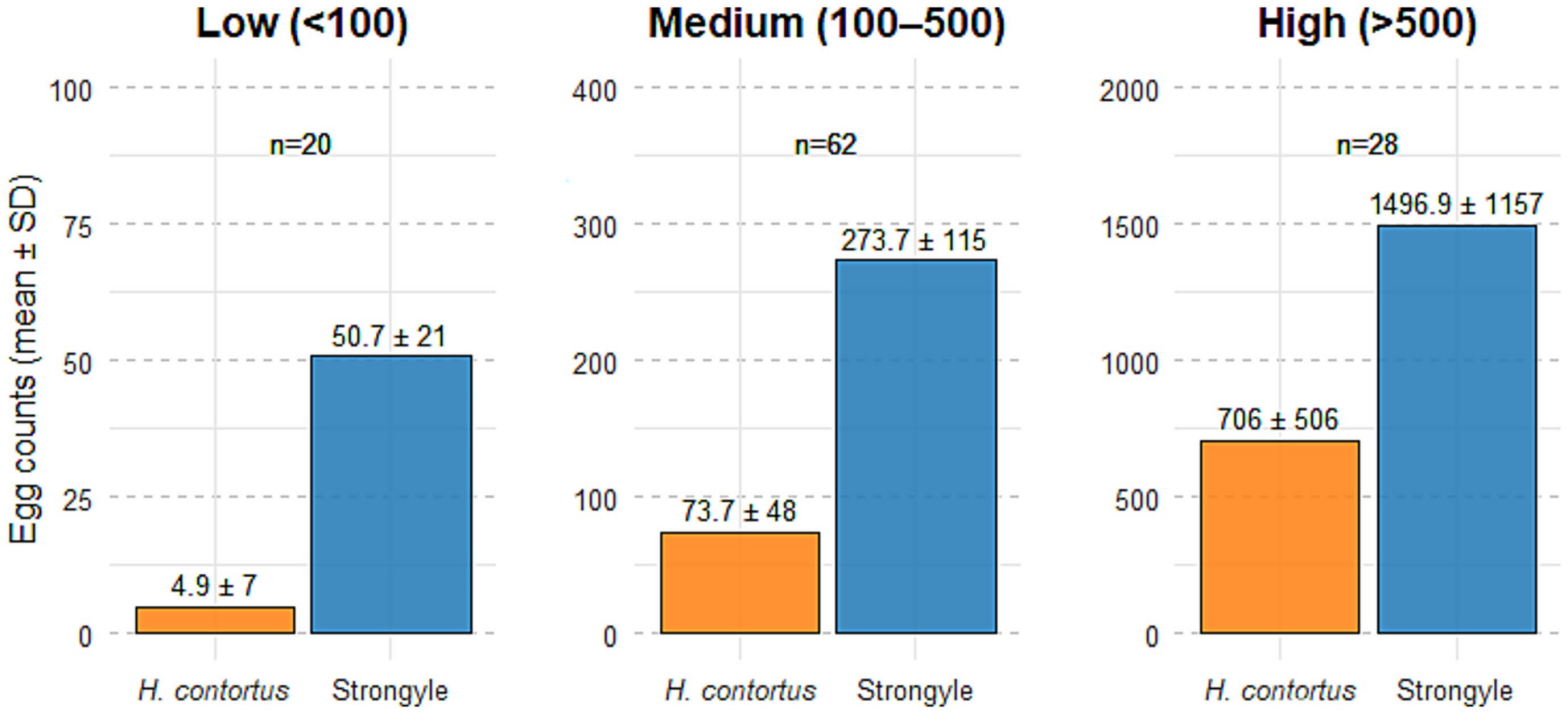
Mean (± SD) egg counts of *Haemonchus contortus* and strongyle-type eggs across three OvaCyte™ strongyle egg count categories: Low (<100 epg), medium (100–500 epg) and high (>500 epg). Sample sizes (n) are indicated above each bar.

Of the 110 ovine faecal samples examined, all (100%) were positive for strongyle-type eggs. Among these, 48% were positive for *Nematodirus* spp., 15% for *Moniezia* spp., 29% for *Strongyloides* spp. and 36% for coccidia. Of the samples positive for strongyle-type eggs, 83.6% (*n* = 92) were positive for *H. contortus*, while 16.4% (*n* = 18) were negative for this species. Based on the results of the OvaCyte™ Speciation strongyle egg counts, the samples were stratified into three categories: low (<100 eggs), medium (100–500 eggs) and high (>500 eggs), Figure 1 and Table 3.

**Table 3:** Summary of OvaCyte™ Speciation strongyle egg count categories, including corresponding sample numbers, mean and ± standard deviation (SD) of strongyle and *H. contortus* egg counts and prevalence data.

OvaCyte™ strongyle egg counts category	No. of samples (%)	Mean (±SD) strongyle egg count	Mean (±SD) *H. contortus* egg count	Mean % *H. contortus* egg count	% *H. contortus* < 50 epg
Low (<100)	20 (18%)	50.7 (±21)	4.9 (±7)	9%	9%
Medium (100–500)	62 (55.8%)	273.7 (±115)	73.7 (±48)	28%	6.45%
High (>500)	28 (25.2%)	1496.9 (±1,157)	706 (±506)	50%	0.00%

### 3.1 Correlation between the PNA and OvaCyte™ Speciation methods

**Figure 2:**
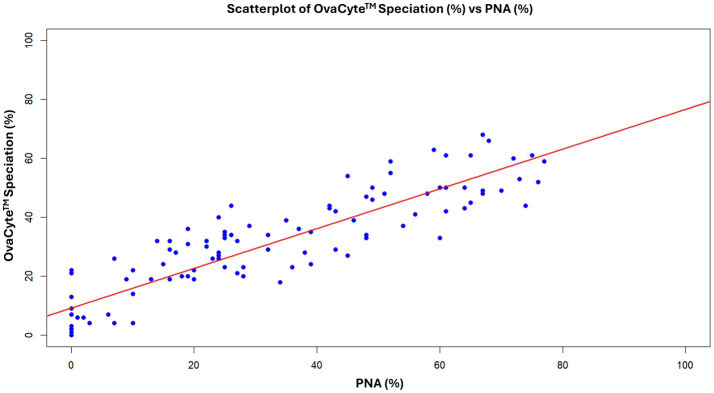
Spearman rank correlation between OvaCyte™ Speciation and peanut agglutinin (PNA) methods for estimating the proportion of *H. contortus* eggs among strongyle eggs in ovine faecal samples.

**Figure 3:**
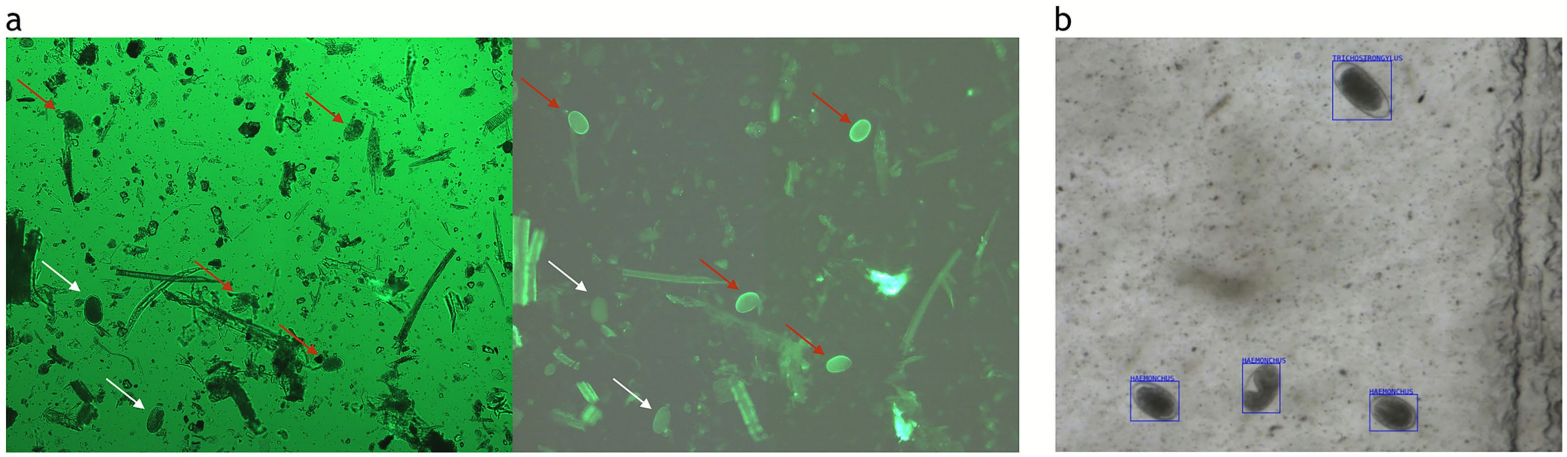
**(a)** Microscopy images of *H. contortus* (red arrow) and other strongyle eggs (white arrow) captured with (left) and without (right) UV light at 100 × magnification using a Nikon pE-300 fluorescent microscope (FITC filter, UK). **(b)** Scanned image of *Trichostrongylus* and *H. contortus* eggs from OvaCyte™ Speciation, showing bounding boxes around detected eggs.

The correlation between the PNA and OvaCyte Speciation methods is presented in Figure 2. The analysis revealed a very strong, statistically significant positive monotonic relationship between the two methods (*rₛ* = 0.90, *p* < 0.05). Figure 3 shows microscopy images of *H. contortus* contortus (red arrow) and other strongyle eggs (white arrow) captured with (left) and without (right) UV light at 100 × magnification using a Nikon pE-300 fluorescent microscope (FITC filter, United Kingdom). (b) Scanned image of *Trichostrongylus* and *H. contortus* eggs from OvaCyte™ Speciation, showing bounding boxes around detected eggs.

### 3.2 Diagnostic performance of OvaCyte™ Speciation for the detection of *Haemonchus contortus*

**Figure 4:**
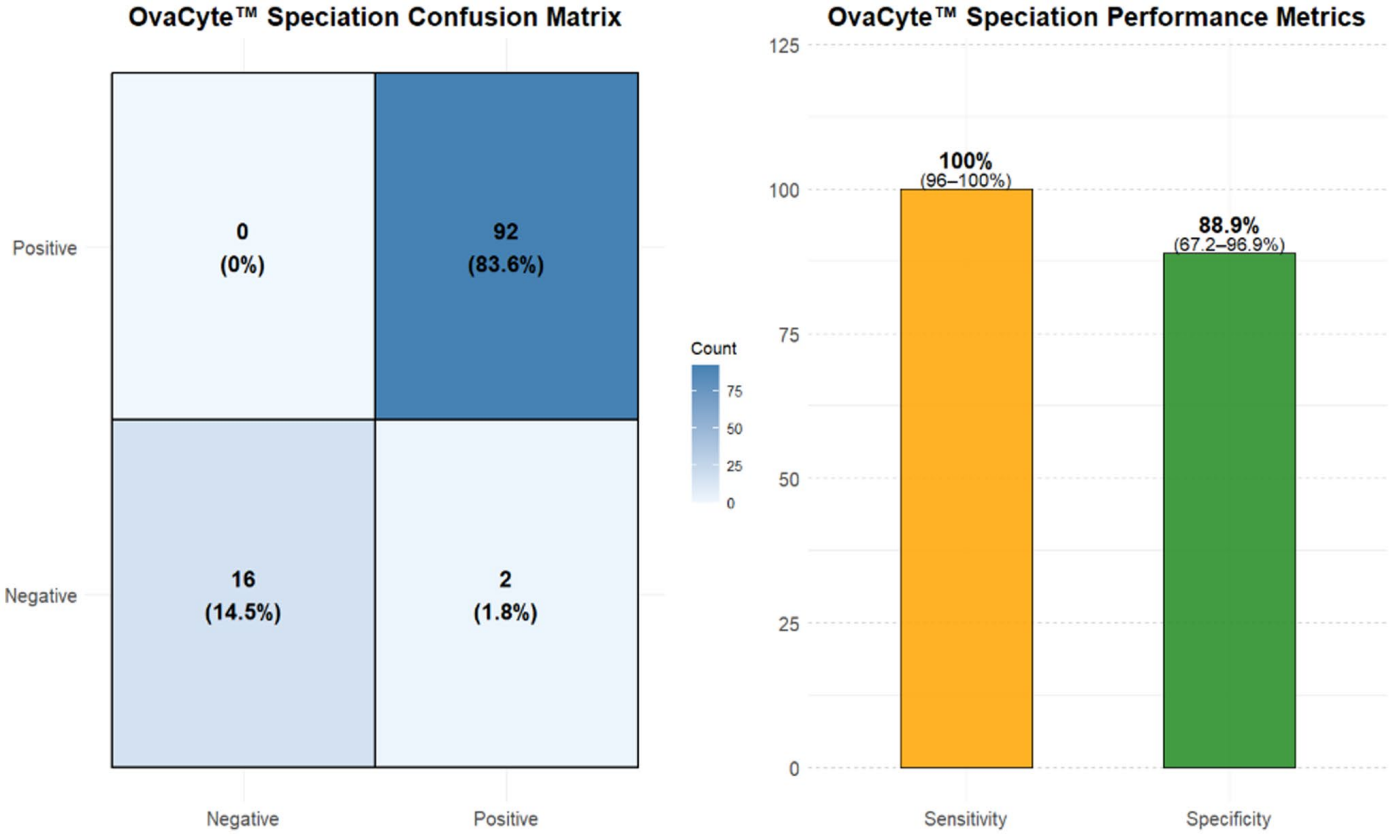
Combined visualisation of OvaCyte™ Speciation test performance. Left: Confusion matrix displays classification results with counts and percentages: true positives (92, 83.6%), true negatives (16, 14.5%), false positives (2, 1.8%) and false negatives (0, 0%). Right: Performance metrics, including 100% Sensitivity and 88.9% Specificity.

The results of the confusion matrix and diagnostic metrics for OvaCyte™ Speciation are summarised in Figure 4. OvaCyte™ Speciation demonstrated high diagnostic accuracy for *H. contortus* detection, with 100% sensitivity (95% CI: 96–100%) and 89% specificity (95% CI: 65–98%).

## 4 Discussion

Automated diagnostic technologies such as OvaCyte™ Speciation offer a significant advancement in the development of sustainable parasite control programmes (14, 15).

This study evaluated the diagnostic utility of the OvaCyte™ Speciation method in estimating *H. contortus* proportions in ovine faecal samples and compared its performance with the PNA fluorescence method, a recognised standard (6). The close alignment of the PNA method with results from coproculture, light microscopy, PCR and loop-mediated isothermal amplification, as demonstrated in multiple studies (12, 19–21), further supports its validity as a reliable reference method.

To understand the relationship between the overall strongyle egg count and the specific proportion of *H. contortus*, the positive samples were grouped into different levels of infection, as indicated in Table 3. The results clearly demonstrated that as the total strongyle egg count increased, the proportion of *H. contortus* rose from a mean of 9% in the low egg count category to 28% in the medium egg count category and ultimately to 50% in the high egg count category. This is to be expected, as the very high fecundity of *H. contortus* results in a high egg count in a positive sample over a short period of time (5). This critical finding indicates that heavy strongyle infections in this population, where mean egg counts reached approximately 1,500 eggs, were primarily driven by *H. contortus.* However, variation within each infection category likely reflects differences in host age, immunity, pasture contamination levels and sample heterogeneity, which are expected under field conditions.

Supporting this, recent data from 2022 reported by the Animal and Plant Health Agency, which offered free *H. contortus* testing using the PNA method, found that 207 of 256 farm submissions (81%) were positive for *H. contortus*. Among the positive samples, the average proportion of *H. contortus* eggs was approximately 40% (22). The ability to quantify this species-specific contribution is crucial, not only for informing treatment decisions but also for designing targeted control programmes that prioritise the most pathogenic and drug-resistant nematode species such as *H. contortus* (23, 24).

Previous studies have questioned the reliability of morphology-based methods for estimating the proportion of *H. contortus* within mixed strongyle populations, particularly due to the overlap in egg morphology among trichostrongylid species (5). These concerns have led to a preference for molecular diagnostics, such as PCR, or species-specific staining techniques like PNA, which offer higher specificity for *H. contortus* identification and quantification. More recent research may challenge this assumption. The comparative study by Ljungström et al. (25) evaluating manual morphological speciation against PCR-based identification, demonstrated a surprisingly high level of agreement between the two methods. This indicates that, with sufficient training and quality control, morphology-based identification could provide reliable results (26, 27), although molecular methods are still generally considered more accurate.

This finding has practical implications, especially in field or resource-limited settings, where molecular techniques may be cost-prohibitive or logistically challenging to implement. In this context, the OvaCyte™ Speciation method, leveraging AI to standardise and scale morphological assessments, may bridge the gap between traditional and molecular diagnostics. By reducing inter-observer variability and increasing throughput, OvaCyte™ Speciation has the potential to offer a practical, cost-effective tool for routine surveillance and targeted control of *H. contortus*.

The value of quantitative methods also needs to be recognised. As faecal egg count reduction tests become a key cornerstone in resistance monitoring, accessible methods of testing that can support the uptake are critical. Low detection limits, high quantitative accuracy and repeatability are also important attributes of such a system (26).

The very strong correlation (*rₛ* = 0.90) between the OvaCyte™ Speciation and PNA methods highlights the reliability of the AI-based OvaCyte™ Speciation method. Despite slight discrepancies at high egg burdens, OvaCyte™ Speciation achieved 100% sensitivity and 89% specificity, supporting its use as a robust and practical diagnostic tool.

At high egg counts (>1,000 epg), the PNA fluorescence method tended to report higher proportions of *H. contortus* compared to the OvaCyte™ Speciation system. This divergence likely reflects methodological differences and inherent limitations in both diagnostic approaches. In OvaCyte™ Speciation, overlapping eggs in heavily infected samples can hinder accurate morphological classification, potentially leading to an underestimation of *H. contortus* proportions. Continued refinement and training of the AI algorithm may help address these challenges. Conversely, the PNA method may overestimate *H. contortus* prevalence in samples with high egg burdens. This is likely due to obscured strongyle counts under bright-field microscopy caused by debris and egg crowding, while fluorescently labelled *H. contortus* eggs remain visible, artificially inflating the species’ relative proportion.

At lower counts (<50 epg), there is a sampling bias in the PNA method due to small subsampling, which may limit its reliability as a gold standard. This is evident in the two false positive (FP) samples for *H. contortus* identified by OvaCyte™ Speciation, both of which had low strongyle counts and <100 epg *H. contortus*. Egg loss during extraction is likely, and egg recovery using the PNA method was notably lower, particularly in low-count samples such as these, where only ~ 50% of strongyle eggs were recovered from the whole aliquot. Therefore, the FP samples may have tested positive with a more sensitive method. Notably, had a threshold of 100 epg been used to define a positive result, both samples would have been reclassified as true negatives. These methodological effects likely explain the small discrepancies, which are not expected to impact clinical decisions significantly. While the PNA method is well-established and offers high specificity, it is time-consuming, resource-intensive and unsuitable for field use. Moreover, it may overestimate *H. contortus* proportions.

The broader benefits of the OvaCyte™ Speciation platform are particularly significant in the context of *H contortus* epidemiology. Hyperacute and acute infections caused by *H. contortus* can lead to severe clinical manifestations and mortality within a matter of days in sheep and goats. Diagnostic delays, particularly when samples are submitted to reference laboratories, may result in missed treatment windows and substantial losses (28). The ability of OvaCyte™ Speciation to provide rapid, species-level identification enables timely, targeted intervention with mono-drug anthelmintic therapy, thereby reducing reliance on broad-spectrum or multidrug approaches that accelerate resistance development. In addition, the use of OvaCyte™ Speciation for screening newly introduced animals facilitates the implementation of effective quarantine and biosecurity protocols, thereby reducing the risk of introducing *H. contortus* into *H. contortus-*free holdings (1, 5, 29). Recent investigations have also explored novel biological and plant-derived control strategies against *H. contortus*, highlighting the continuing global relevance of effective diagnostic tools (23, 30, 31).

A limitation of this study is the absence of samples derived from animals infected exclusively with *H. contortus*. In natural field settings, mono-infections are highly uncommon, as gastrointestinal nematode infections typically occur in mixed populations. Consequently, definitive mono-infected samples can usually only be obtained through controlled experimental infections, which were beyond the scope of this study.

## 5 Conclusion

This study supports the OvaCyte™ Speciation platform as a reliable, scalable and field-adaptable alternative to PNA fluorescence microscopy for the proportional estimation of *H. contortus* in ovine faecal samples. The very strong concordance observed between OvaCyte™ Speciation and the PNA method, particularly in moderate to high infection burdens, demonstrates its practical diagnostic value, especially in clinical and field settings where rapid results, minimal training and operational efficiency are essential. Although PNA fluorescence microscopy remains a valid reference method, its resource-intensive nature limits its routine applicability. OvaCyte™ Speciation addresses these limitations and provides a promising tool for routine surveillance and targeted treatment. Future iterations of the platform may incorporate cloud-based data integration and multi-species diagnostic models to support herd-level surveillance and precision parasite control.

## Data Availability

The original contributions presented in the study are included in the article/supplementary material, further inquiries can be directed to the corresponding authors.
